# Addressing unresolved tensions to build effective partnerships: lessons from an Aboriginal cancer support network

**DOI:** 10.1186/s12939-015-0259-7

**Published:** 2015-11-04

**Authors:** Beatriz Cuesta-Briand, Dawn Bessarab, Shaouli Shahid, Sandra C. Thompson

**Affiliations:** Western Australian Centre for Rural Health, University of Western Australia, UWA, M706, 35 Stirling Highway, Crawley, WA 6009 Australia; Centre for Aboriginal Medical and Dental Health, UWA, Perth, Australia

**Keywords:** Aboriginal Australians, cancer services, Collaboration, Indigenous, Partnership, Perspectives, Support groups, Worldviews

## Abstract

**Background:**

Cancer is the second leading cause of death among Aboriginal and Torres Strait Islander people and their survival once diagnosed with cancer is lower compared to that of other Australians. This highlights the need to improve cancer-related health services for Indigenous Australians although how to achieve this remains unclear. Cancer support groups provide emotional and practical support, foster a sense of community and belonging and can improve health outcomes. However, despite evidence on their positive effects on people affected by cancer, there is scarce information on the function and effectiveness of Indigenous-specific cancer peer-support programs in Australia.

Using qualitative data from an evaluation study, this paper explores different understandings of how a cancer support group should operate and the impact of unresolved tensions following the establishment of an Indigenous women cancer peer-support network in a regional town in Western Australia.

**Methods:**

Data were collected through semi-structured interviews with 24 participants purposively selected among Indigenous and mainstream healthcare service providers, and group members and clients. Interviews were audiotaped and transcribed verbatim. Transcripts were subjected to inductive thematic analysis. NVivo was used to manage the data and assist in the data analysis. Rigour was enhanced through team member checking, coding validation and peer debriefing.

**Results:**

Flexibility and a resistance to formal structuring were at the core of how the group operated. It was acknowledged that the network partly owned its success to its fluid approach; however, most mainstream healthcare service providers believed that a more structured approach was needed for the group to be sustainable. This was seen as acting in opposition to the flexible, organic approach considered necessary to adequately respond to Indigenous women’s needs. At the core of these tensions were opposing perspectives on the constructs of ‘structure’ and ‘flexibility’ between Indigenous and non-Indigenous participants.

**Conclusions:**

Despite the group’s achievements, unresolved tensions between opposing perspectives on how a support group should operate negatively impacted on the working relationship between the group and mainstream service providers, and posed a threat to the Network’s sustainability. Our results support the need to acknowledge and address different perspectives and world views in order to build strong, effective partnerships between service providers and Indigenous communities.

## Background

Aboriginal and Torres Strait Islander (hereafter Indigenous^1^) Australians have higher rates of preventable cancers than other Australians [1]. Despite some improvements in cancer detection in recent years, Indigenous people are still experiencing delayed diagnoses, and, once diagnosed, they have poorer outcomes than other Australians [1, 2]. Compared with other Australians, Indigenous people were more likely to be diagnosed with cancer at a younger age; have a higher incidence of rapidly fatal cancers (lung, liver) and a lower incidence of cancers with better survival [3]. Indigenous Australians diagnosed with cancer between 1999 and 2007 had a 40 % chance of surviving for at least 5 years, compared to non-Indigenous Australians (52 %). In 2007–2011, the age standardised cancer mortality rate was 1.5 times higher for Indigenous Australians than for non-Indigenous Australians (252 compared with 172 per 100,000) [AIHW, 2013]. Lower access to cancer screening programs and deficiencies in treatment [1], and cultural barriers including fatalism, shame and preference for traditional healing [4] contribute to these poor outcomes. In addition, communication issues between Aboriginal patients and cancer service providers [5, 6] further impede engagement of Indigenous cancer patients in non-Indigenous (mainstream) services.

Cancer support groups provide a type of support that is distinct from the support offered by other supportive relationships [7]. Systematic reviews of peer-support programs for people with cancer found that peer support groups provided benefits to participants, including receiving emotional and practical support [8], and experiencing a sense of comfort and camaraderie from speaking with someone who shares similar experiences [8, 9]. Qualitative research conducted in Australia shows that support groups provide a sense of community, unconditional acceptance and information, while at the same time facilitating positive relationships with family and friends [7]. Evidence from an exploration of Aboriginal women with breast cancer in Canada suggests that mainstream support groups may not meet the needs of Aboriginal women [10], and in Australia, Indigenous cancer patients are not accessing mainstream support groups and are poorly represented among clients accessing cancer support services overall [11].

Despite the positive effects of support groups on people affected by cancer, there is scarce evidence on Indigenous-specific cancer peer support programs in Australia, with an Indigenous Women’s Cancer Action Group operating in South Australia providing one of the few examples [11]. A model for Indigenous peer mentoring developed in Melbourne showed that a consultative and responsive approach was essential, and the development of partnerships with Indigenous communities and mainstream services and agencies was critical [12]. This is consistent with findings from a literature review of partnerships between Indigenous and mainstream health services [13], and with an environmental scan of the Cancer Councils (state-based peak cancer organisations in Australia), which highlighted challenges in building and sustaining collaboration with Indigenous organisations [11]. Overall, there is a dearth of information on how Indigenous cancer clients can be better supported through their cancer journey by culturally-appropriate cancer support groups and whether this might be one way of reducing the disparities in cancer treatment and outcomes that Indigenous Australians experience.

This paper uses qualitative data from an evaluation of an Indigenous cancer support network (the Network) operating in a regional town located approximately 400 km from Perth, Western Australia. The regional city has a population of around 35,000 people and supports services across a large region in which the major industries are agriculture, fishing, mining and human services. Around 11 % of the regional population is Aboriginal. The Network was started with a paid part-time coordinator with support from peer volunteers, and held fortnightly meetings at a community-based venue. The data collection occurred over a period of time, beginning after the network had been operating for around nine months, and had provided emotional and practical support to an estimated 50 women affected by cancer, including women undergoing cancer treatment, cancer survivors and carers [14].

Preliminary descriptive results have been reported elsewhere [15]. This paper presents results from the in-depth analysis of the qualitative data collected and focuses on the data relating to unresolved tensions between different Indigenous and mainstream perspectives on how a support group should operate.

## Methods

This research was part of a study investigating cancer experiences, attitudes and beliefs among Indigenous Australians. Ethics approval for the study was granted by the Western Australian Aboriginal Health and Ethics Committee, and Curtin University Human Research Ethics Committee (HR121/2009), and all participants provided informed consent.

### Sample and data collection

A purposive sampling technique [16] was used to recruit participants to the study; the sample was recruited among key informants from Indigenous and mainstream health service providers operating in the region, and from Network members and clients. Interviews were undertaken separately on two occasions with key IWCSN members and key cancer service providers focusing on what was working well in their collaborative working relationship and what needed to be improved. Participants were invited to share their views on the role and effectiveness of the Network through open-ended questions such as ‘What do you think the main achievements of the Network have been?’ or ‘What are the challenges the Network has faced?’ and What could change to make it work better?

Participants were also brought together formally at a workshop forum to further discuss issues raised and to focus on ways of addressing areas needing improvement. A particular focus of the forums was to ask both Aboriginal and non-Aboriginal participants how their cultural conditioning/learning may have impacted on concerns raised about their working partnership; whether understanding of their relevant cultural perspectives is likely to be helpful in the ongoing development of this partnership; and how they could acknowledge these respective concerns and move to working more effectively in assisting Indigenous women to access cancer screening and treatment.

### Data analysis

Sessions were audio-recorded and transcribed verbatim. Interview transcripts were subjected to thematic analysis. The analysis was largely inductive and a special emphasis was placed on comparing and contrasting participants’ views, and exploring areas of tension. The data analysis process broadly followed the steps described by Green and colleagues [17]: immersion in the data, coding, creating categories and identifying themes.

NVivo 10 [18] was used to manage the data and assist in the data analysis; in particular, the ‘Model’ feature was used to visually map connections between coding categories [19]. Using NVivo facilitated collaboration between research team members and enhanced rigour by adding transparency to the data analysis process [20]. Furthermore, rigour was enhanced through team member checking, coding validation and peer debriefing [21].

## Results

A total of 24 participants (female, *n* = 22; male, *n* = 2) took part in the study. The sample included: Indigenous workers and representatives from Indigenous healthcare service providers (*n* = 3); representatives from mainstream cancer services and agencies (*n* = 8); Network members (*n* = 6); and Network clients (*n* = 4). In addition, one group interview was conducted with three Indigenous women who had not had contact with the network. The sample reflected the diversity of stakeholders involved and yielded rich data that allowed for the in-depth exploration of the topic.

Two main areas of tension were identified which influenced participants’ perceptions of the strengths and weakness of the Network: the tension between structure and flexibility relating to the operations of the Network, and different understandings of the notion of confidentiality. These tensions are discussed separately and are illustrated with quotes that are contextualised by the use of the interview identifier and the description of the stakeholder group represented.

### The tension structure versus flexibility

All stakeholders acknowledged that the group was still ‘finding their feet’ and evolving, and although it was perceived that the Network partly owned its success to its ‘organic’ and ‘fluid’ approach, most mainstream service providers believed that a more structured approach would be needed for the Network to be sustainable. References to the absence of structure featured frequently in participants’ accounts, and elicited strong, opposing reactions from Indigenous and non-Indigenous participants.

The tension between Indigenous and mainstream perspectives on the need for structure was underpinned by opposing understandings of the constructs of ‘structure’ (also conceptualised as ‘terms of reference’ or ‘guidelines’) and ‘flexibility’. As seen in Table 1, Indigenous participants tended to associate the construct of ‘structure’ with notions which had negative connotations for them, such as bureaucracy, government control and the ‘whitefella way’. On the other hand, a flexible approach was associated with attributes such as ‘grassroots’, ‘holistic’, ‘community-controlled’, ‘laidback’ and ‘organic’, all of which had positive connotations and were perceived to be at the core of the Network’s success.

**Table 1:** Indigenous and mainstream perspectives on the constructs of ‘structure’ and ‘flexibility’

Construct	Indigenous perspective	Mainstream perspective
*Structure (terms of reference, guidelines)*	Bureaucracy	Confidentiality
	Whitefella way	Clinical safety
	Government language	Sustainability
	Government control	Clarification of roles
	Formality	Boundaries
		Clarity
		Transparency
*Flexibility (absence of terms of reference or guidelines)*	Laid-back	Confidentiality issues
	Holistic	Clinical safety issues
	Grassroots	Sustainability issues
	Community-controlled	Lack of transparency
	Organic	
	Inclusive	
	Relaxed	

In contrast, most mainstream service providers tended to discuss the absence of structure in the context of sustainability and confidentiality issues, and clinical safety, and they called for the development of terms of reference, which in their view would bring transparency and clarity for all stakeholders, including Network members, clients, and service providers.

**Fig. 1:**
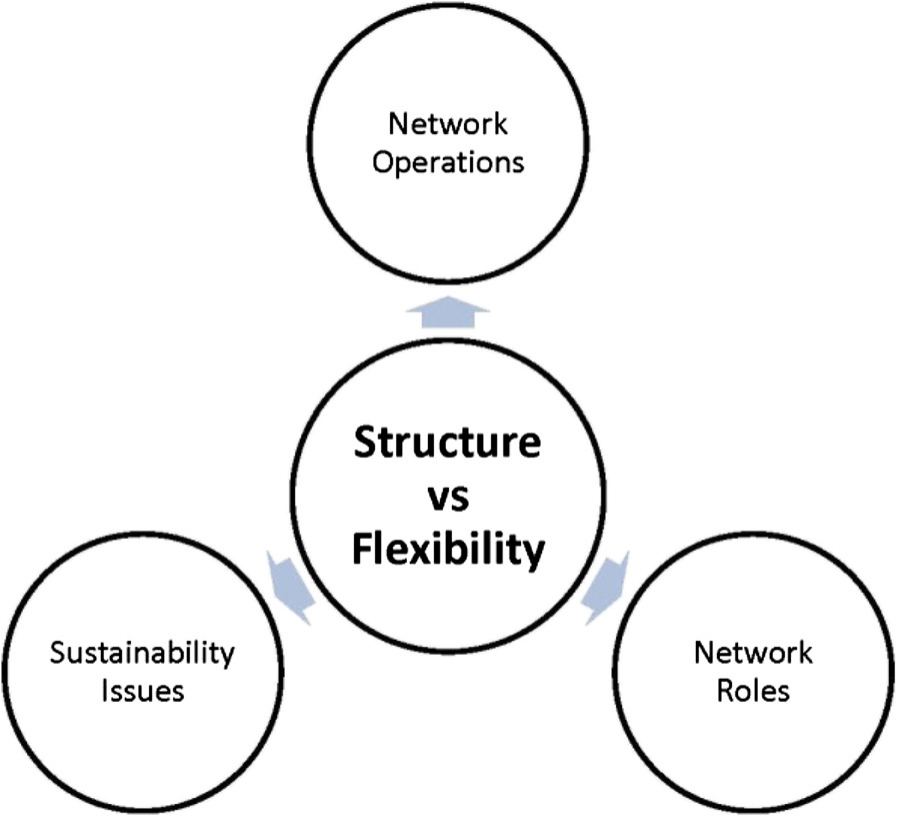
Diagram representing the domains affected as a result of the tension between cancer Network members and mainstream service providers over Network structure and flexibility

As seen in Fig. 1, tensions between contrasting attitudes towards the need for structure shaped stakeholders’ views on the roles and operations of the Network, and influenced their perceptions of the Network’s sustainability issues.

#### Network operations

Flexibility and lack of formality were at the core of how the Network operated. While these attributes were perceived as critical to the Network’s success by Indigenous stakeholders, they triggered concerns among mainstream service providers.

The Network provided information, and practical and emotional support to Indigenous women affected by cancer individually at home and as a group at the Network’s fortnightly meetings. The meetings were held at a community venue and were attended by Network members and clients; local service providers were also invited to attend. These sessions provided an opportunity for Network members to report back to the group on their activities in the community, and for women to get together and experience the comfort of listening to and learning from other women going through similar journeys. Despite having an agenda, the meetings were fluid and activities varied depending on the dynamics of the group on a particular day. *Yarning* (an Indigenous style of informal conversation and storytelling) was seen as an essential component of these meetings, fostering a relaxed, laid-back atmosphere, which in turn created a culturally-safe space for women to connect with other women and with service providers. One Indigenous service provider explained the healing power of yarning and as she compared this approach with other ‘rigid’ approaches, her account highlighted the issue of operating to specific timelines, a characteristic that Indigenous stakeholders associated with mainstream service provision and perceived as a barrier to Indigenous engagement:

*Yarning breaks down so many barriers, and the more you get to yarn and the more you get to meet that person, the more they give of themselves to you. If you come in all stiff and all rigid and you have got a set thing and you have got timelines, deadlines, and you have got other people and other things to do, they are going to know that. So, therefore, they are going to say, ‘Okay, I am only going to give you that much of me’. So all you will get is just the answers to your questions. You will never get a part of that person’s life. You won’t get a part of their soul, because they will go, ‘Well, you are not really interested. You are here to do this and you have made it clear that you have only got this amount of time, so, no’.* (01, Indigenous service provider)

Mainstream service providers who had attended the meetings agreed that the sessions seemed to have a positive impact on women attending. One mainstream service provider believed that the ‘magic’ of the group came from that fluidity and not knowing who was going to be at the meetings, adding that the Network accumulated a wealth of knowledge because of the diversity of the group, as the women had been exposed to every facet of caring. In contrast, another service provider said she needed to know where she fitted ‘in the scheme of things’ when attending these meeting, adding:

*It is difficult when you are looking at a mixed group of people that have people that are at different stages in the cancer journey and also people who don’t have a diagnosis of cancer but are keen to help and be involved. So yeah, they all come with different agendas.* (07, mainstream service provider)

Service providers spoke about the need for facilitation and supervision of the meetings, and, overall, their accounts suggested that they had some reservations about the fluid way in which the sessions were conducted, although they were reluctant to criticise the Network. One provider who had worked closely with the Indigenous community for many years acknowledged that she was ‘comfortable with that sort of organic approach’ although she believed that others in her position would find it more difficult. This service provider thought that some structure was needed, but ‘a broad framework rather than a rigid prescription’; later, she reflected:

*The difficult things that need the most sort of support end up with the least because they don’t fit the mould*. (08, mainstream service provider)

There was a shared recognition that the Network largely owed its success to its being a grassroots initiative, and that the Network’s achievements were a testament to the strength, passion and commitment of its members. Indigenous participants felt strongly that the power of the Network came from being a community initiative led by caring and passionate Indigenous women – from women ‘speaking and learning from the heart’.

While Indigenous participants perceived commitment and passion as positive attributes which underpinned the Network’s operations, these attributes elicited mixed reactions from mainstream service providers. Although mainstream stakeholders acknowledged that passion and commitment had contributed to the Network’s achievements, they tended to discuss ‘passion’ and ‘enthusiasm’ in the context of the need for boundaries, clinical supervision and preventing burnout, and their accounts suggested that they believed these attributes needed to be reined in rather than nurtured. Thus, ‘passion’ tended to have negative connotations, and was perceived as a liability rather than an asset. The following account from one service provider encapsulates this view whilst highlighting other underlying tensions:

*It is also important that you are not emotionally driven and demanding, because a couple of times there has been an absolute ‘This is happening now, this is a crisis. You need to come’ or ‘We need to see you’. I can’t sustain that. Like everybody else, you wait three weeks for a doctor. I am not that important, but I have got lots of clients and I am often pre-booked, and I can’t just respond on that minute. So that makes it difficult too. So, yeah, it is about understanding, I guess, about how the health services run.* (03, mainstream service provider)

The mainstream health service saw her role as providing care in the context of a serious illness, but this was not a crisis service and hence she had a different understanding regarding what they should and could respond to in any given moment.

#### Network roles

Different perceptions were observed among participants with regard to the Network’s roles in the community, and there was some evidence of tension between these contrasting views. These tensions related to the broad role of the Network in the community, and also to specific services provided by the Network. Mainstream service providers perceived that a lack of clarity about purpose and roles had been an issue from the Network’s inception and was now a major sustainability issue. In the accounts of most service providers, the sustainability of the Network depended on having terms of reference which would set boundaries around what services would and would not be provided. References to ‘confusion’ and ‘lack of clarity’ punctuated the accounts of mainstream service providers when they discussed the Network’s roles, and they often referred to the notion of clinical safety when calling for boundaries and clarification of roles, explaining that they needed to feel ‘safe’ that their clients were going to be ‘safe’ if they were referred to the Network. One service provider explained:

*I would be really happy with some operational guidelines so that I can be clear telling the patient what I am referring them for, say, practical support, and ‘if you do want peer support, there is that option, but let’s get it clear. ‘If you have got kids to pick up from school, this is where the group can help you’.* (03, mainstream service provider)

There was evidence that the perceived lack of role definition and boundaries had led to some service providers not referring clients to the Network, and some questioned the Network’s ability to support their clients until such issues were resolved. Overall, mainstream service providers held the view that the Network needed to define its ‘core business’ and establish clearly defined roles.

In contrast, Indigenous participants had a broad understanding of the role of the Network, and believed that the Network had a role to play in ‘breaking down barriers across all service providers’, extending their reach to women affected by conditions other than cancer and their families. It was widely perceived that the Network would not ‘turn their back’ on anybody seeking their support; a comment by a Network member reflected this attitude, and shed light on the nature of the some of the issues Indigenous women faced in the community:

*I feel it is getting out to them and reaching them and bringing them in and meeting other women with these problems, but not only cancer. They come and talk about all the other problems, whether their child has been molested or they have been abused. That all ties in when we get together as a women’s group*. (13, Network member)

In the context of role definition, the establishment of boundaries had negative connotations for Indigenous participants, who perceived this as curtailing their ability to help families in the community. Structure was perceived as being in opposition to the fluid and organic approach required to adequately respond to Indigenous women’s needs. It was believed that adopting a formal, structured approach would narrow the focus of their work and decrease their ability to address women’s complex psychosocial issues, including financial stress, domestic violence and housing issues. This strong negative reaction towards the establishment of ‘structure’ is encapsulated by this comment from an Indigenous service provider:

*You put a structure up and the thing about structures is they like to be very clear about what the role and the function is and what the purpose of the reference group is, whereas this way it is a global work and they can touch on any and everything.* […] *There are enough Aboriginal organisations out there that operate under structure. If you want structure, go to them. That is where mainstream are so hell bent on control all of the time, and that is why little initiatives such as this die.* (01, Indigenous service provider)

With regard to the emotional support provided by the Network, different understandings of how a support group should operate aggravated the unresolved tensions around role definition. Service providers involved in providing psychosocial support to cancer patients voiced concerns about a perceived lack of volunteer training and supervision, and their accounts suggested that they had trouble reconciling the fluid way in which the Network operated with their experience of mainstream support group facilitation. One service provider said that she needed to feel safe in the knowledge that she was referring patients to a safe environment, adding:

*If it is focused and patient-driven, then surely we can work around and make a plan to make all parties safe, and work within those boundaries but keep their individuality, you know.* (11, mainstream service provider)

Lastly, mainstream service providers voiced concerns relating to practical support services provided by the Network which had their root in a perceived lack of clarification of roles. Referred to as the ‘behind the scenes’ work, the Network had been involved in providing home support services such as cleaning and gardening, driving women to their medical appointments, and picking up and dropping off children at school. Several service providers raised concerns about the Network’s involvement in practical support service delivery and the potential for liability issues should a volunteer be injured or involved in a car accident while performing their volunteering work.

#### Sustainability issues

The tension between structured and fluid approaches also underpinned participants’ perceptions of the Network’s sustainability issues. A perceived lack of support from mainstream service providers and securing on-going funding were the main sustainability issues for Indigenous stakeholders. In contrast, mainstream service providers believed the risk of burnout was as a major sustainability issue. There was a shared perception among all stakeholders that the Network’s success was a result of the passion and commitment showed by the core group of women who were involved in running the Network – especially the coordinator and a handful of Network volunteers. On the one hand, all agreed that the passion and energy of these women were driving the Network; on the other hand, many were concerned that Network members were at risk of burnout because they were ‘giving too much’, and working with women who had complex health and psychosocial problems only aggravated this risk. Supervision, mentoring and adequate support were seen as critical to harness the passion and energy of the women, and the need for the women to engage in self-care was identified, arguing that ‘it is okay not to give all the time’. One service provider spoke about this issue:

*Because they are motivated and they are passionate, and everybody thinks that is wonderful, which it is, but that sort of behaviour can often manifest itself in over-commitment and, you know, being too harshly judging of themselves in they don’t achieve something and that sort of thing. So they need supporting and very closely someone to mentor them and somewhere where they can debrief them, really to be kept on track in some ways about caring for themselves.* (07, mainstream service provider)

As a result, mainstream service providers perceived lack of structure as a major sustainability issue in itself. Although service providers acknowledged that some flexibility was needed, the data shows that some found it challenging to reconcile this notion with the perceived need for a more structured approach. Equally, there was widespread concern that formalising the group ‘too much’ would turn women away. References to adopting a more structured approach elicited strong negative reactions from Indigenous participants, who firmly believed that formalising the group would lead to its downfall. Reflecting on the organic way in which the Network had operated, the Network coordinator explained:

*If we have got to go and start structuring it too much, those women are going to turn around and say: ‘It is getting too much whitefella way now’ and they are going to back off.* (12, Network member)

Similarly, one Indigenous service provider described the formalisation of the Network as her ‘greatest fear’, adding:

*The minute you start talking about structuring, terms of reference, policies, procedures, little groups like this fall part. […] You start talking about putting in structures… forget it!*

Later in the interview, she reflected:

*The minute you start doing that and governments start putting structures in place and all those things, and then the carrot that the Government tends to dangle at these types of initiatives is money, that then creates all sorts of problems. The power in the group is that they are grassroots women from the community who are speaking and learning from the heart.* (01, Indigenous service provider)

The quote above is illuminating as it brings together two issues, setting up structures and funding, which were seen as the main sustainability issues by Indigenous participants and mainstream service providers respectively. This quote also highlights the importance of grassroots initiative and how to support a bottom-up approach.

### Tensions between different perspectives on confidentiality

The analysis of the data revealed that although all stakeholders valued confidentiality highly, different perspectives on the notion of confidentiality and its practical implications had led to unresolved tensions which resulted in a lack of trust between Indigenous and mainstream stakeholders.

Tensions around confidentiality came to the fore when discussing the way in which women were referred or linked in with the Network. Referral processes were fluid and largely done through word-of-mouth (this form of communication was referred to as ‘grapevine’ or ‘bush telegraph’). One woman provided an account that was representative of the experiences of other Network clients. She described how she had come into contact with the group:

*Through family and friends. They were out speaking with my Aunty [name], and they advised [the Network coordinator] of my condition and then, yeah, we made contact. I don’t know if she contacted me or what. Yeah, I think it probably happened that way, yeah.* (19, Network client)

Although mainstream service providers acknowledged that operating through the ‘bush telegraph’ was an effective way of reaching out to women in the community, many thought that a more formalised process was needed, citing potential for confidentiality issues and lack of transparency as the main reasons. Two alleged breaches of confidentiality were reported by mainstream service providers, and although they were partly attributed to the Network ‘finding its feet’; it was evident that these incidents had had a negative impact on the working relationship between the Network and service providers. In both cases, the Network was seen to have become involved against the wishes of the cancer patient involved. One service provider spoke about one woman with a poor prognosis and complex psychosocial issues feeling ‘very challenged’ by the Network’s involvement, explaining:

*This particular [patient] felt that she couldn’t say that she didn’t want any help from that group or that her parents shouldn’t be seeing them because it was disrespectful, because one of the group was related somewhere or were very good friends of her mum, or her mum’s cousin.* (03, mainstream service provider)

The service provider explained that this woman did not want her relatives involved, and the service provided had respected this wish; however, this created a misunderstanding as the Network perceived that there was lack of action on the service provider’s part. Referring to another woman who had declined the offer to be referred to the Network, this service provider explained:

*The assumption that everybody needs support even within European culture is not necessarily what the patient wants at the time. It might come later, but you introduce it. But there is an assumption that because someone is Indigenous that you will immediately refer just because of their Aboriginality. If I have done an assessment on somebody and really asked them if they would like to be linked and they say ‘no’, I am not going to contravene that.* (03, mainstream service provider)

There was a perception among service providers that the bush telegraph may be limited to family connections, and this was seen to be aggravated by the fact that the Network operated in a small community where social and family connections were tight. Thus, service providers called for the establishment of referral mechanism which, in their view, would add transparency to the referral process. In addition, service providers placed emphasis on personal choice and patient consent, and believed that referral processes needed to respect the woman’s choice to decide whether to access the Network or not. One service provider spoke of their concerns about confidentiality issues:

*‘I can’t put my finger on it, but there is something in the back of my mind that says, ‘Have we got it right yet?’. Whether it is the people or whether it is the structure, I am not sure. That might be the nurse in me talking, because we are big on confidentiality*. (04, mainstream service provider)

The quote above refers to being ‘big on confidentiality’; this was a common argument among service providers, many of whom currently worked or had worked in the past for the Health Department and were used to working within strict guidelines around patient privacy and confidentiality. Similarly, another service provider explained:

*I need to be safe when I am referring that they are going to be safe. Confidentiality is of the utmost importance, and that I am not going to meet a health professional or a volunteer member from the group in the shopping centre and they are going to start to talk to me about a client. I have had that experience and feel very uncomfortable and very unsafe for myself, but, most importantly, for the client.* (11, mainstream service provider)

It must be noted that the incident reported by the mainstream service provider above was unrelated to the Network; it had, however, an impact on the service provider’s perception of potential confidentiality issues. The account is also representative of a perception among most mainstream service providers that Indigenous people may not value confidentiality highly; one service provider, however, pointed out:

*Aboriginal people value their privacy I think more than white people.* (08, mainstream service provider)

Both privacy and confidentiality were highly valued by Indigenous participants, and respecting women’s privacy and the wishes of those who ‘want to be by themselves’ underpinned the way in which Network members initiated contact with women in the community. The Network itself was seen as a space where privacy and confidentiality would be protected, as Network members reported that some Indigenous people would not access local Indigenous health services. One Network member explained:

*You will get some people that may not want to access [Indigenous health service], you know, because they say ‘It is not confidential. It is not the paper side of it. It is because people see them going in, you know, and that is their sort of broken confidentiality*. (15, Network member)

One Network member explained ways in which client confidentiality was protected, for example not including full names on reports, and reminding Network members and clients about the importance of keeping everything that was discussed at the meeting ‘in the room’. She also alluded to the difficulties of maintaining confidentiality in a context of strong community and family connections:

*Every time we have meetings it always comes up, ‘What is said in this room is confidential and it stays in this room’. Because with Aboriginal people, because a lot of them are always concerned about family and stuff, they might be talking, but we don’t say anything. We try and be professional, to keep, you know, ‘We heard about it’. Like, they come and tell us ‘Did you hear about such-and-such?’ and we say ‘Yeah’, but that is about as far as we go. We don’t go into any details of things.* (14, Network member)

One comment by a Network member who reflected on a conversation she had had with a mainstream service provider about the misperception that Indigenous people do not respect confidentiality encapsulates the tensions between Indigenous and mainstream service providers:

*It seems to be their [service providers’] sort of confidentiality side of things. I don’t know what it is. I said, you know, ‘just because you think we are a blackfella service, it doesn’t mean we are not confidential. Everything is highly confidential here. Everything is locked up at the end of the day, all the filing cabinets, you know’. […] but Wadjelas seem to have a thing about blackfellas, ‘they tell someone something and they go and tell that, and they will go and tell that, and it will get around that grapevine and everyone knows’. So it is really hard to break that cycle with them*. (15, Network member)

The quote above highlights tensions between Indigenous and mainstream perspectives and points to the challenge of ‘breaking the cycle’ and build effective partnerships based on trust and respect.

## Discussion

The Network operated in a regional town in Western Australia and provided support to Indigenous women affected by cancer and their families. The Network was widely acknowledged to have been successful in connecting families with cancer needs with existing services. The Network was perceived to act as a ‘cultural broker’ [6], providing a culturally safe space for women to engage with cancer services and with health promotion and screening initiatives. Somewhat in contrast with findings from Shahid and colleagues [5], the regional mainstream service providers interviewed had a sound understanding of the life circumstances of Indigenous patients, and they acknowledged that the current model of care in which they operated failed to address the complex health and psychosocial needs of Indigenous cancer patients and their families. There is evidence that patient treatment models that address the social, cultural and treatment needs of Indigenous patients can improve treatment compliance [22]. The Network was perceived as bridging the gap in cancer service delivery and, in this context, its multidimensional role might be compared to that of a cancer navigator, which has been suggested as an effective strategy to support Indigenous women diagnosed with cancer [10]. A substantial difference with models of navigator programs that are well established and have proved successful in the United States is the commitment to training of community or peer navigators that occurs there [23, 24].

Fluidity and flexibility characterised the operations of the Network. Although this finding is consistent with other evidence on culturally appropriate Indigenous peer-support groups [12], it was found to be a source of conflict in this study. Opposing perspectives on the constructs of ‘structured’ and ‘flexible’ were observed between Indigenous and non-Indigenous participants. ‘Structured’ tended to have positive connotations for mainstream service providers, while the construct tended to elicit negative reactions from Indigenous participants. This unresolved tension negatively impacted on the working relationship between the Network and service providers, and highlights the need for Indigenous organisations and mainstream service providers to work through different perspectives on service delivery, building trusting relationships over time [13].

In this study, the main issue affecting effective collaboration related to process/structure factors [25], more specifically, the perceived lack of clear role and operational guidelines. In addition, there was some evidence of tension between different understandings of patient confidentiality. This finding is consistent with research exploring the practice of confidentiality in an Indigenous medical service which found that because of the complexity of the role of Indigenous health workers and their place in the community, staff made judgements on which information to use and which not to use in the healthcare setting [26].

There is need for strong, effective partnerships between services providers and agencies to increase quality of life and survival rates for Indigenous people in regional areas [11]. Sharing power and resources is important in helping sustain any partnership [27]. It proved difficult for mainstream health professionals with many years of training to accept the different approach adopted by volunteer support group members despite evidence that they were successful at engaging Indigenous community members with cancer and their families. The experience suggests that there is a need for volunteer training and support for such Indigenous support groups. Examination of peer navigator models operating elsewhere suggests ways for how support and training may be provided [24, 28].

For initiatives such as this Indigenous cancer support Network to be effective and sustainable, tensions between different perspectives on service delivery must be addressed and resolved. Two-way learning and working, based on professional trust and respect offers a way forward. Availability of formal training and mentoring relationships needs to be explored.

## Conclusions

Despite goodwill of both the Indigenous Women’s Cancer Support group and mainstream service providers, unresolved tensions limited the partnership. Mainstream providers recognised that the Network was achieving in areas where they had struggled given their longstanding issues of effectively engaging Indigenous clients. However, their concerns about the lack of structure, ironically seen as integral to the effective functioning of the group, and lack of professional boundaries were of sufficient concern to limit their participation and referral to the Network. In turn, this impacted upon the achievements and sustainability of the group which was a concern that was shared by both Indigenous and non-Indigenous interviewees. Understanding such tensions and learning to work better in partnership is one essential component of efforts to improve the inequities in cancer outcomes experienced by Indigenous Australians.

There needs to be recognition in policy of the important role that volunteers play in bridging community and health services and greater support to help develop, support and sustain for Aboriginal leadership in this area. The value community leaders provide partly derives from the fact that they are less restricted in the way that they operate than mainstream service providers who may be straightjacketed by risk management and organisational protocols which constrain flexibility in meeting patient needs. Understanding this experience and how to work better across the interface of professional health services and Indigenous communities is essential to expediting better Indigenous cancer and other health outcomes.

## Footnotes

1 The Indigenous people of Australia are Aboriginal and Torres Strait Islanders. While in general, the local Indigenous people in Western Australia prefer to be called Aboriginal, the Women’s Cancer Support group which is the subject of this research preferred to us the term Indigenous because they wished to be inclusive. Hence, we respectfully use the term Indigenous throughout this paper, except where the word Aboriginal was used within an included quotation or as a proper noun.
